# Unveiling KLHL23 as a key immune regulator in hepatocellular carcinoma through integrated analysis

**DOI:** 10.18632/aging.206167

**Published:** 2024-12-04

**Authors:** Liangliang Xu, Bo Li, Yuchen Liu, Zhengming Hu, Qing Dan, Bingxuan Xu, Hongjin Xiang, Yun Chen, Tingting Zheng, Desheng Sun, Li Liu

**Affiliations:** 1 Shenzhen Key Laboratory for Drug Addiction and Medication Safety, Department of Ultrasound, Institute of Ultrasonic Medicine, Peking University Shenzhen Hospital, Shenzhen Peking University-The Hong Kong University of Science and Technology Medical Center, Shenzhen 518036, China China; 2 Department of Clinical Oncology, The University of Hong Kong-Shenzhen Hospital, Shenzhen 518053, China; 3 Guangdong-Hong Kong-Macau University Joint Laboratory of Digestive Cancer Research, Scientific Research Center, The Seventh Affiliated Hospital, Sun Yat-Sen University, Shenzhen 518107, China; 4 Guangdong Provincial Key Laboratory of Digestive Cancer Research, Scientific Research Center, The Seventh Affiliated Hospital of Sun Yat-Sen University, Shenzhen 518107, China

**Keywords:** pan-cancer analysis, KLHL23, hepatocellular carcinoma, prognosis, invasion and metastasis

## Abstract

Age-related cancers are characterized by impaired protein homeostasis, where Kelch protein superfamily members have showed accumulating clues as critical regulators. In this paper, the cancerous role of Kelch-like family member 23 (KLHL23) was comprehensively analyzed with TCGA and single cell GEO database across overall 33 cancer types. By multi-omics analysis upon the transcriptomic, genomic, and methylation data, the current study explored the association of KLHL23 with patient survival, gene ontology, tumor-infiltrating lymphocytes, and drug responses. The correlation of copy number variations and methylation with dysregulated expression of KLHL23 were also addressed. Notably, KLHL23 levels correlated with survival in cancers such as hepatocellular carcinoma and low-grade glioma. The study also highlighted how reduced KLHL23 expression is linked to increased immune activity and sensitivity to chemotherapy, suggesting its potential as a biomarker for cancer prognosis and treatment responsiveness.

## INTRODUCTION

Kelch-like family member 23 (KLHL23) is a gene encoding a member of the Kelch-like protein family [1]. These proteins are characterized by their Kelch motifs, which are composed of multiple β-propeller domains, and typically play multiple roles in various cellular processes, including regulation of protein homeostasis which correlated to aging and cancer [2]. The KLHL23 gene is located on chromosome 7 and is part of the larger family of Kelch-like genes, which are known for their involvement in the ubiquitin-proteasome system. This system is of paramount importance for protein degradation and turnover within the cell, thus ensuring the maintenance of cellular protein homeostasis [3, 4]. The precise functions of KLHL23 remain to be elucidated, yet its potential role in cancer biology is a subject of ongoing investigation [5, 6]. Changes in the expression or mutation of the KLHL23 gene may have implications for cell proliferation, apoptosis, and other dysregulated pathways during oncogenesis. Comprehensive study of KLHL23 within the context of different cancer types can provide insights into its relation to tumor development and progression and potentially reveal novel therapeutic targets [7].

Recent studies have shown that KLHL23 is crucial in the process of the epithelial-mesenchymal transition (EMT) in tumorigenesis [7, 8]. KLHL23 suppresses the EMT in liver cancer cells by obstructing their ability to reorganize the actin cytoskeleton. Consequently, actin is identified as the sole unique E3 substrate for KLHL23 [2]. While, the regulation effect of KLHL23 across human cancers remains largely unknown, and its role in tumor microenvironment needs further studies [9]. Here, we conducted a pan-cancer analysis using The Cancer Genome Atlas (TCGA) database to investigate its potential role in cancer. We analyzed the expression of KLHL23 in different tumors, examined how its expression correlated with patient’s outcomes and overall survival rates, and investigated how its genetic or epigenetic alterations might contribute to cancer development. Furthermore, we used single cell sequencing data to evaluate our findings. We also examined various factors, including gene expression, survival status, DNA methylation, genetic alterations, immune infiltration, and relevant cellular pathways, to elucidate the possible molecular mechanisms of KLHL23 in human cancers. In addition, a drug sensitivity analysis of KLHL23 was used to predict more personalized treatment strategies by assessing individual tumor responses to specific drugs.

## MATERIALS AND METHODS

### Data collection and processing

The comprehensive sequencing data were obtained from TCGA portal [10], supplemented by normal tissue sample data from the GTEx website [11]. The TCGA repository has cataloged over 20,000 primary cancer samples along with matched non-cancer samples from 33 different cancer types. We integrated both the RNA sequencing datasets and the associated clinical annotations. Using the “rma” function within the R software environment (version 4.2.2), we extended the dataset by removing instances of missing or duplicate data. A log2(TPM+1) transformation was then applied to the expression data, where TPM is transcripted per million. For the comparative analysis of KLHL23 gene expression levels in cancer and normal samples, we extracted relevant gene expression data from the 33 cancer types profiled in TCGA to construct an expression matrix. We also retrieved detailed patient tumor and clinical staging information and additional clinical data from the same portals.

### Cox regression analysis and survival analysis

This study employed Cox proportional hazards regression analysis to evaluate the prognostic value of KLHL23 for overall survival (OS), disease-specific survival (DSS), disease-free interval (DFI), and progression-free interval (PFI) across various cancers, utilizing the log2-transformed TPM expression values of the gene obtained from the TCGA database. OS was defined as the time from the date of diagnosis to the date of death from any cause. DSS considered only deaths that were directly attributable to the disease and excluded other causes. PFI accounted for either disease progression or death from any cause, while DFI was limited to instances of disease progression.

We generated survival curves for distinct cancer types by categorizing patients into groups with high or low expression of KLHL23 using the median expression level as the dividing threshold, employing the Kaplan-Meier method. These analyses were conducted using the “survivalROC” and “survival” packages in R [12, 13]. Differences between the survival curves were evaluated using a log-rank test, with a significance threshold set at a *P*-value of less than 0.05. Subsequently, we created a forest plot to visualize the association between survival and KLHL23 expression, and conducted Kaplan-Meier analyses to compare the OS among TCGA cancer patients grouped by median expression levels of KLHL23, utilizing the log-rank test for difference comparison between lower and higher KLHL23 expression groups.

For extended survival analysis of KLHL23 across various cancer types, we referred to the PrognoScan database, which consolidates survival data from numerous Gene Expression Omnibus (GEO) datasets. This provided a broader context for the implications of KLHL23 expression levels in cancer prognosis.

### Immune cell infiltration

In our methodology, the estimation of stromal and immune cells in malignant tumor tissues using expression data (ESTIMATE) algorithm was used to infer the cellular composition of cancer samples based on their transcriptional profiles [14]. This algorithm computes three distinct scores via single sample gene set enrichment analysis (ssGSEA): the stromal score, indicating of the presence of stromal cells within the tumor; the immune score, reflecting the extent of immune cell infiltration in the tumor environment; and the estimate score, which suggests the overall tumor purity [15]. By applying the ESTIMATE algorithm to transcriptional data, we ascertained both immune and stromal scores for the cancer tissues.

Subsequently, we explored the relationship between these scores and the expression of KLHL23. In an effort to delve deeply, we utilized CIBERSORT, an advanced metagene-based tool that accurately quantifies 22 distinct human immune cell phenotypes, to assess the link between KLHL23 expression and each immune cell phenotype across the 33 types of cancer [16]. Additionally, we investigated how KLHL23 levels correlated with markers of tumor-infiltrating immune cells.

Correlation analyses were performed using Spearman’s correlation coefficient to determine statistical significance. The outcome of these analyses was represented visually through an expression heat map for each corresponding gene-immune cell marker pair within each cancer type studied.

### Gene set enrichment analyses

To examine the relationships between KLHL23 and other genes, we performed correlation analyses with data sourced from TCGA. Pearson’s correlation coefficients were employed to assess the strength and direction of the associations between KLHL23 and other genes in the dataset. Only genes that demonstrated a significant correlation with KLHL23 (*P*-value < 0.05) were considered for further analysis using gene set enrichment analysis (GSEA).

GSEA utilized the Molecular Signatures Database (MSigDB) and tools from the Bioconductor project, as well as the R software environment for statistical computing [17]. Enrichment maps were used to visually represent the gene sets enriched in our analysis. These maps facilitated understanding of gene expression patterns and the potential pathways involved in biological processes associated with KLHL23.

### Drug response analysis

To explore the correlation between KLHL23 expression and patient response to chemotherapy in different cancer types, we used the pRRophetic package [18]. This package uses a ridge regression model that incorporates baseline gene expression levels along with drug sensitivity data derived from a variety of cell lines. This innovative methodology allowed us to predict clinical outcomes in response to chemotherapeutic agents based on the individualized gene expression profiles of patients prior to treatment.

### Statistical methodology

In our survival analysis, we constructed Kaplan-Meier survival curves, performed log-rank tests for group comparisons, and used the Cox proportional hazards model. In addition, we examined more nuanced associations using Spearman’s rank correlation. R software, version 4.2.2, was used for all statistical analyses. We reported our data as means accompanied by standard deviations. Student’s *t*-tests were used for comparisons between two different groups. In accordance with standard practice, we considered results to be statistically significant if the *P*-value was less than 0.05.

## RESULTS

### KLHL23 pan-cancer expression analysis

**Figure 1:**
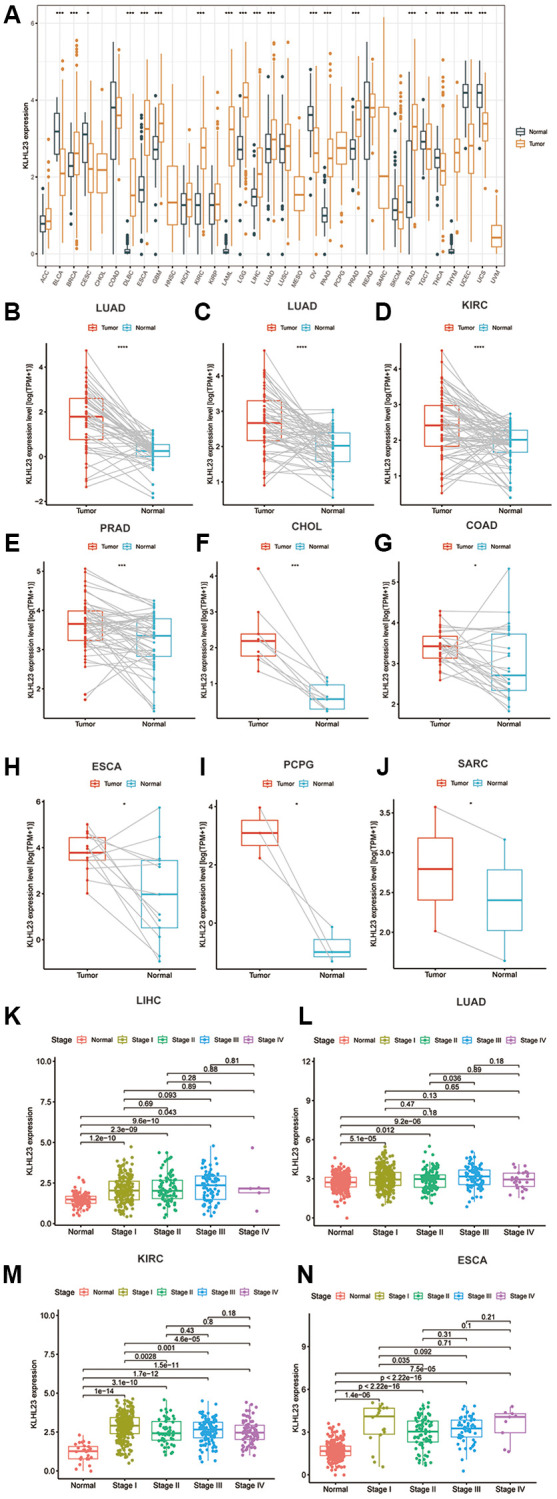
**The differential expression of KLHL23 in human tumors and healthy tissues among the 33 types of cancer.** (**A**) The black and yellow bar graphs indicate KLHL23 levels in normal and tumor tissue. (**B**–**J**) Pan-cancer differential expression of KLHL23 in paired tumor and adjacent normal tissue in the indicated, represents tumor types (liver hepatocellular carcinoma (LIHC), LUAD, KIRC, PRAD, CHOL, COAD, ESCA, PCPG, and SARC) from the TCGA database. (**K**–**N**) KLHL23 mRNA expression levels in diverse stages of the indicated tumor types from the TCGA database (LIHC, LUAD, KIRC, ESCA). Blue and red bars indicate normal and tumor tissue KLHL23 levels (^*^P < 0.05, ^**^P < 0.01, ^***^P < 0.001).

To investigate the expression patterns of KLHL23 in various cancer types, a comprehensive analysis of KLHL23 in 33 different cancer types in the TCGA dataset was conducted. Our findings revealed a significant upregulation among 14 types of cancer in comparison to their corresponding normal tissue (Figure 1A), including breast invasive carcinoma (BRCA), diffuse large B-cell lymphoma (DLBC), esophageal carcinoma (ESCA), glioblastoma multiforme (GBM), clear cell renal cell carcinoma (KIRC), acute myeloid leukemia (LAML), lower-grade glioma (LGG), liver hepatocellular carcinoma (LIHC), lung adenocarcinoma (LUAD), pancreatic adenocarcinoma (PAAD), prostate adenocarcinoma (PRAD), stomach adenocarcinoma (STAD), thymoma (THYM), and uterine carcinosarcoma (UCS). Conversely, lower levels of KLHL23 expression were observed in five types of cancer when compared to normal tissue: bladder urothelial carcinoma (BLCA), ovarian cancer (OV), testicular germ cell tumors (TGCT), thyroid carcinoma (THCA), and uterine corpus endometrial carcinoma (UCEC) (Figure 1A). The differential expression analysis of KLHL23 may prove beneficial in the development of diagnostic tools for the early detection of cancer.

For further confirmation of the upregulation among 14 cancer types above, we conducted mean comparison on matched normal and tumor tissues, and the results showed significant paired upregulation in LIHC, LUAD, KIRC, PRAD, cholangiocarcinoma (CHOL), colon adenocarcinoma (COAD), ESCA, pheochromocytoma and paraganglioma (PCPG), and sarcoma (SARC). These results highlight KLHL23 as a potential biomarker in various cancer types (Figure 1B–1J).

Additionally, we investigated the relationship between KLHL23 expression and cancer stage. Our findings indicated that KLHL23 expression levels were elevated in advanced stages of several cancers, including LIHC, LUAD, KIRC, and ESCA (Figure 1K–1N).

### Somatic mutation and copy number variation characteristics of KLHL23

**Figure 2:**
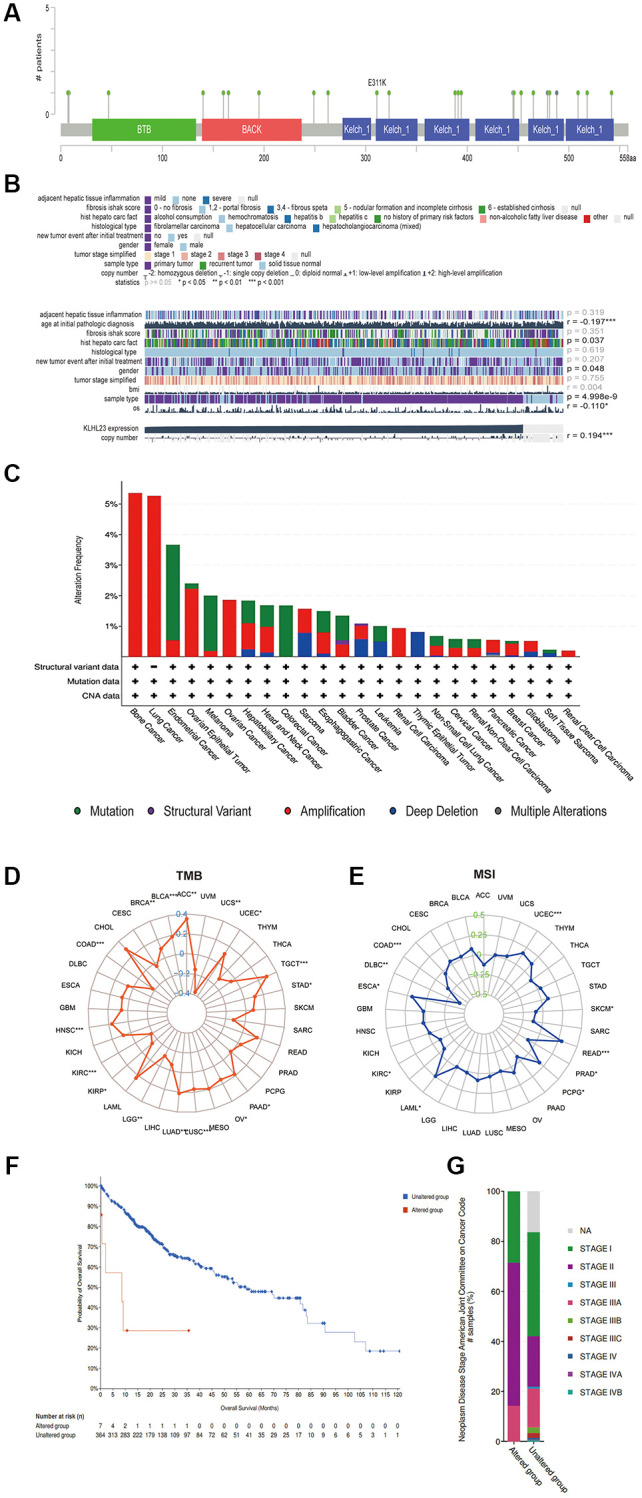
**Somatic mutation and copy number variation characteristics of KLHL23.** (**A**) CNV analysis of KLHL23 in 33 cancer types. (**B**) Mutation sites of KLHL23 as indicted through a cBioPortal analysis. (**C**) Genomic alterations of KLHL23. (**D**) TMB analysis of the indicated cancers. (**E**) MSI analysis of indicated cancers. (**F**) Survival analysis of the altered or unaltered genome of LIHC. (**G**) The altered genome of LIHC according to the cancer stage. (^*^P < 0.05, ^**^P < 0.01, ^***^P < 0.001).

Somatic mutation and copy number variations (CNVs) are the two types of mutations that disrupt chromosomal stability and lead to oncogenic activation or tumor suppressor silencing, play a crucial role in driving tumorigenesis by altering gene dosage and expression patterns. Among these alterations, gene amplification is particularly notable, being most frequent in bone, lung, ovarian, and liver cancers (Figure 2C). Given the upregulated expression of KLHL23 in prevalent solid cancers, we decided to explore the correlation between KLHL23 mutations and expression in LIHC utilizing the Catalogue of Somatic Mutations in Cancer (COSMIC) database. Among the 418 LIHC cancer samples examined, the most prevalent genomic alteration was nucleotide mutation, followed by amplification and deletions. The KLHL23 protein exhibited mutations of E311K in the KELCH domain and T208A in the BTB and C-terminal Kelch (BACK) domain. Within LIHC cancer samples, T208A mutations were most frequently observed (Figure 2A, 2C). Liver cancer samples with mutation in KLHL23 showed higher cancer stage, and those patients had a worse prognosis (Figure 2F–G). Subsequently, we employed Gene Set Cancer Analysis (GSCA) to scrutinize whether a copy number variation (CNV) and DNA methylation in KLHL23 influenced its expression across 33 different cancer types. The results showed a positive association between CNV and KLHL23 expression (Figure 2B). We then analyzed the expression level of KLHL23 with the average DNA methylation levels near promoter region, and the result showed the majority of methylation events were negatively correlated with KLHL23 expression (Figure 2B). Moreover, tumor mutational burden (TMB) and microsatellite instability (MSI) show negative correlation with KLHL23 expression levels in COAD, KIRC, and KIRP (Figure 2D–2G).

### Analysis of the relationship between KLHL23 pan-cancer expression levels and prognosis

**Figure 3:**
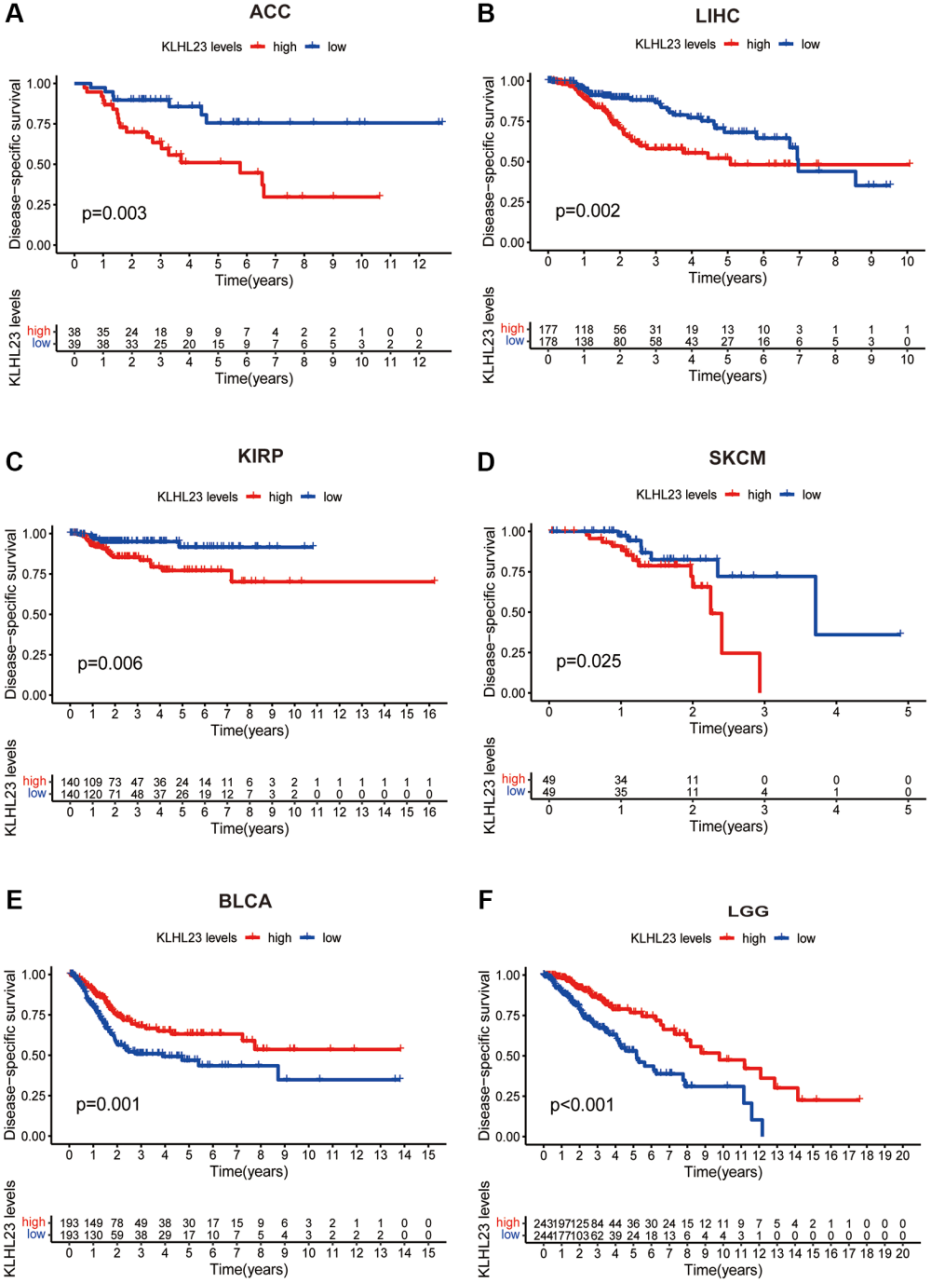
**Association between survival and KLHL23 expression across cancers.** (**A**–**F**) Kaplan-Meier overall survival in indicated representative tumor types according to KLHL23 expression. The median value of KLHL23 in each tumor was used as the cut-off value. (^*^P < 0.05, ^**^P < 0.01, ^***^P < 0.001).

Further analysis was conducted to examine the prognostic significance of KLHL23 expression in various cancer types. Our results showed that high expression of KLHL23 was associated with a poor prognosis in patients with liver cancer (LIHC, *P* = 0.002), adrenocortical carcinoma (ACC, *P* = 0.003), Kidney renal papillary cell carcinoma (KIRP, *P* = 0.006), and skin cutaneous melanoma (SKCM, *P* = 0.025) (Figure 3A–3D). Meanwhile, low expression of KLHL23 was an indication of poor prognosis in patients with BLCA (*P* = 0.001) and LCG (*P* < 0.001) (Figure 3E, 3F).

**Figure 4:**
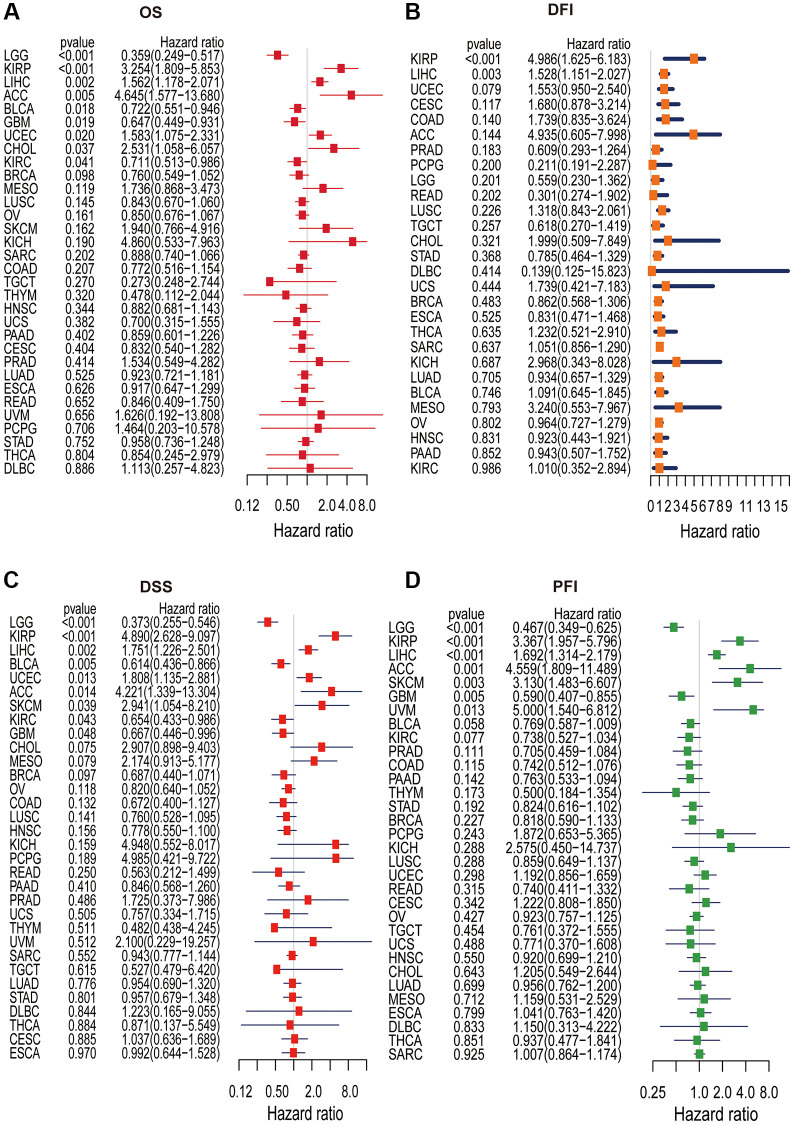
**Univariate Cox regression analysis of KLHL23.** (**A**–**D**) The forest plot shows the relationship between KLHL23 expression and patient overall survival (OS), disease-free interval (DFI), disease-specific survival (DSS), and progression-free interval (PFI). (^*^P < 0.05, ^**^P < 0.01, ^***^P < 0.001).

Single-variate Cox regression analysis was performed to examine the relationship between KLHL23 expression levels and OS, DFI, DSS, and PFI in different cancer types. High KLHL23 expression was found to be a risk factor for OS, DSS, and PFI in many cancer types (Figure 4A–4D). Based on the analysis of OS, elevated KLHL23 expression was identified as a risk factor for KIRP (*P* < 0.001), LIHC (*P* = 0.002), ACC (*P* = 0.005), UCEC (*P* = 0.020), and CHOL (*P* = 0.037). In contrast, lower expression of KLHL23 was associated better overall survival prognosis in LGG (*P* < 0.001), BLCA (*P* = 0.018), GBM (*P* = 0.019), and KIRC (*P* = 0.041).

The Cox analysis results with DFI data showed KLHL23 expression was positively associated with DFI in KIRP (*P* < 0.001) and LIHC (*P* = 0.003). In terms of DSS, high expression of KLHL23 was a risk factor in KIRP (*P* < 0.001), LIHC (*P* = 0.002), UCEC (*P* = 0.013), ACC (*P* = 0.014) and SKCM (*P* = 0.039). Conversely, expression levels of KLHL23 were negatively correlated with DSS in LGG (*P* < 0.001), BLCA (*P* = 0.005), KIRC (*P* = 0.043), and GBM (*P* = 0.048). In terms of PFI, high expression of KLHL23 was identified as a risk factor for KIRP (*P* < 0.001), LIHC (*P* < 0.001), ACC (*P* = 0.001), SKCM (*P* = 0.003), and uveal melanoma (UVM, *P* = 0.013), while lower expression of KLHL23 was found to be a risk factor for LGG and GBM. In summary, the analysis revealed that elevated levels of KLHL23 expression were associated with an increased risk of worse clinical output in LIHC and KIRP, regardless of the OS, DFI, DSS, and PFI.

### The relationships between KLHL23 expression and immune score and stromal score

The ESTIMATE method is a valuable tool in the field of oncology, particularly for assessing the tumor microenvironment based on transcriptional expression profiles. The method uses gene expression data to derive scores that reflect the presence and abundance of stromal and immune cells within the tumor. These scores, known as the immune score and stromal score, are critical for understanding the complex interactions within the tumor microenvironment and their impact on cancer progression and response to therapy.

**Figure 5:**
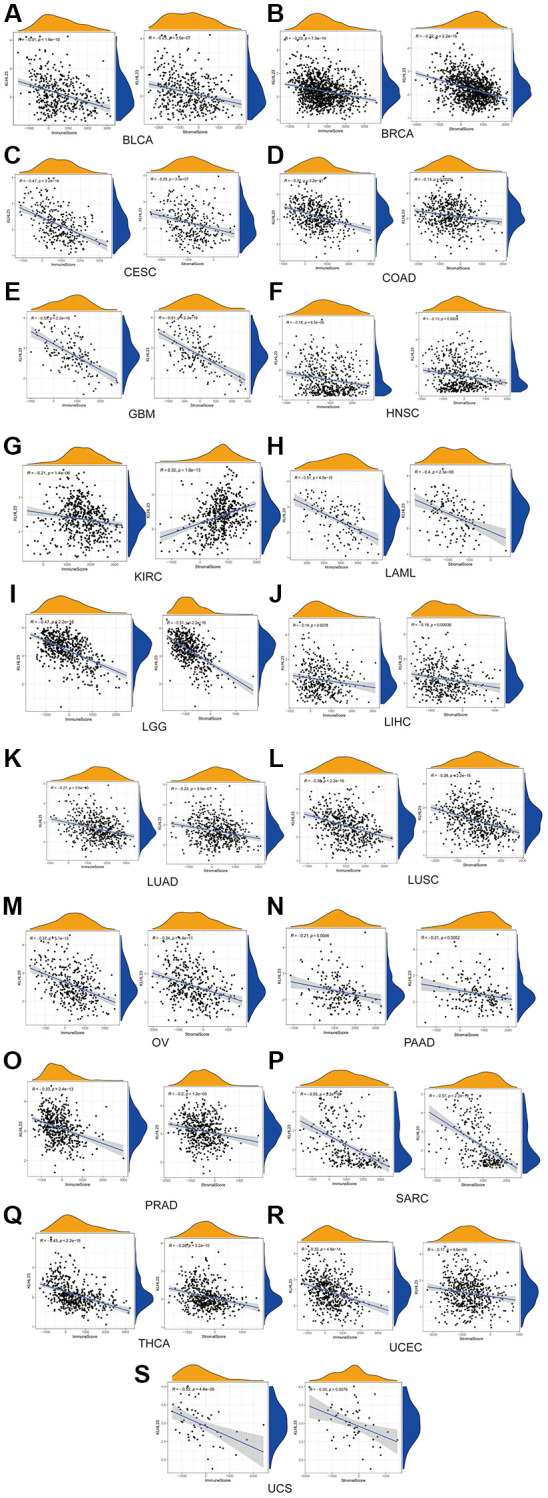
**The negative association between KLHL23 expression and immune score and stromal score.** (**A**–**S**) KLHL23 expression and these scores. Correlation analysis between KLHL23 expression and immune and stromal scores. (R, correlation coefficient; ^*^P < 0.05; ^**^P < 0.01; ^***^P < 0.001; ^****^P < 0.0001).

In our study, a negative association was observed between KLHL23 expression and these scores. Our results suggest that higher levels of KLHL23 expression were linked to reduced immune and stromal cell infiltration within the tumor (Figure 5A–5S). The observed negative correlation between KLHL23 expression and immune/stromal cell infiltration scores highlights the possible involvement of KLHL23 in regulating the tumor microenvironment. The negative correlation between KLHL23 expression and immune/stromal cell infiltration suggests that increased KLHL23 levels could be associated with a less active immune environment within the tumor, which may have implications for the effectiveness of immunotherapy and other treatments relying on the body’s immune response to combat cancer cells. Furthermore, this association highlights the potential role of KLHL23 in modulating the tumor microenvironment and could provide insights into the mechanisms underlying tumor progression and metastasis.

### Association between KLHL23 expression and immune infiltration level in cancer

To analysis the role of KLHL23 for immune regulation, several immune scoring analyses were performed utilizing RNA expression data from The Cancer Genome Atlas (TCGA) database.

**Figure 6:**
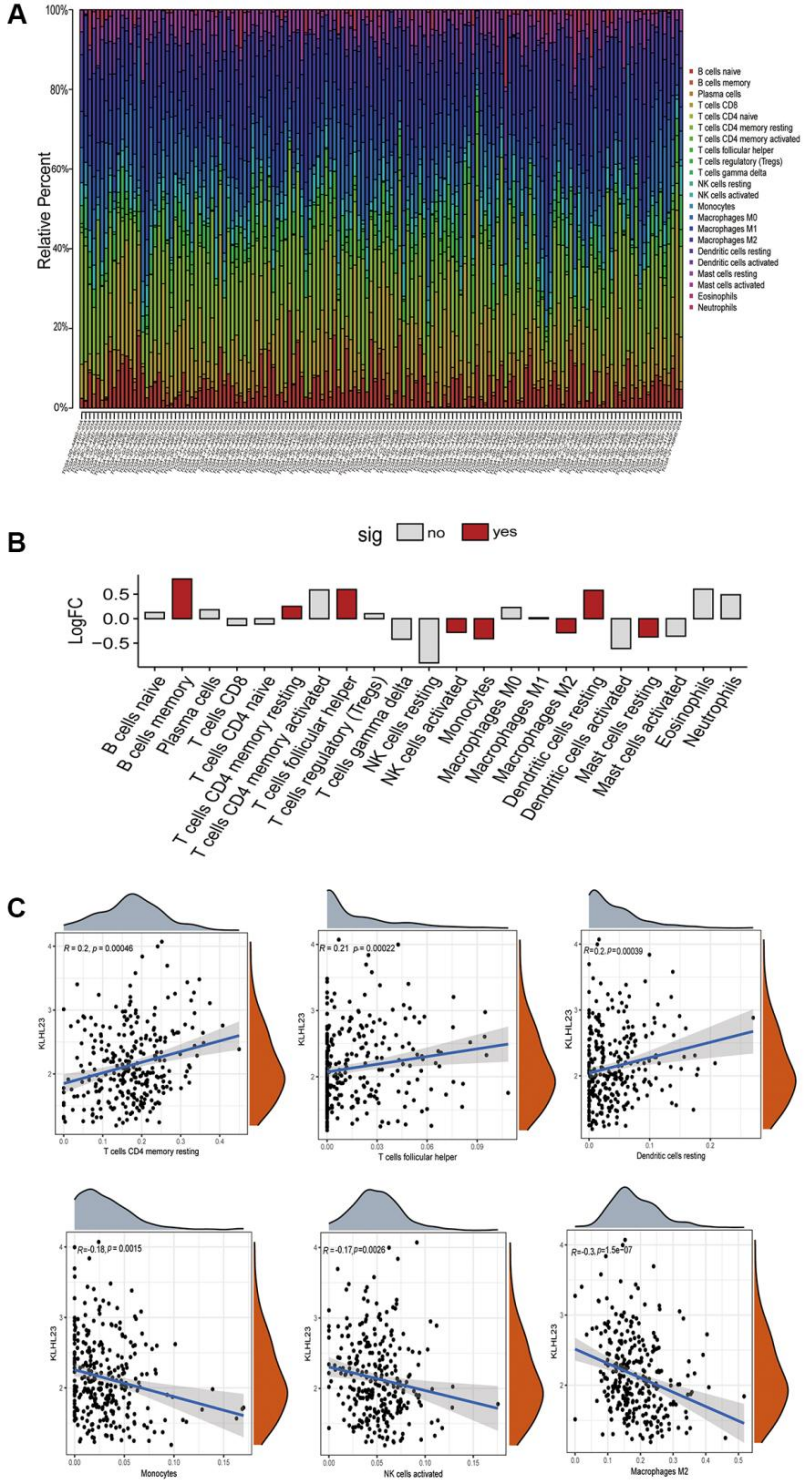
**The role of KLHL23 in the tumor microenvironment of LIHC.** (**A**) Immune cell distribution in KLHL23 high expression LIHC patients. This panel presents a comprehensive analysis of immune cell distribution in the tumor microenvironment of each LIHC patient using data from the TCGA database. The visualization shows the diversity and prevalence of different immune cell types in different patient samples, providing insight into the immune landscape within LIHC tumors. (**B**) Differences in the various immune cell proportions between groups with high and low KLHL23 expression in LIHC. (Abbreviations: no: no significant difference; yes: significant difference). (**C**) KLHL23 expression associated with immune cells. This section illustrates the results of a comprehensive association analysis between KLHL23 expression levels and a wide range of immune cells within LIHC tumors.

The CIBERSORT method was used to determine the distribution pattern of immune cells with high and low KLHL23 expression in each patient sample (Figure 6A and Supplementary Figure 1). In addition, we examined immune cell infiltration and the relationship between each immune cell type and KLHL23 expression. Elevated levels of KLHL23 expression were significantly associated with reduced counts of immune cells in LIHC (Figure 6B, 6C). Higher KLHL23 expression showed a positive correlation with immune cells such as CD4 memory T cells, T follicular helper cells and resting dendritic cells. Conversely, higher KLHL23 expression was negatively correlated with tumor-infiltrating lymphocytes (TILs), including monocytes, activated natural killer (NK) cells, and M2 macrophages. These findings suggest a potential intricate involvement of KLHL23 in the regulation of these key immune cells within the tumor microenvironment.

### Enrichment analysis of KLHL23 in cancers

**Figure 7:**
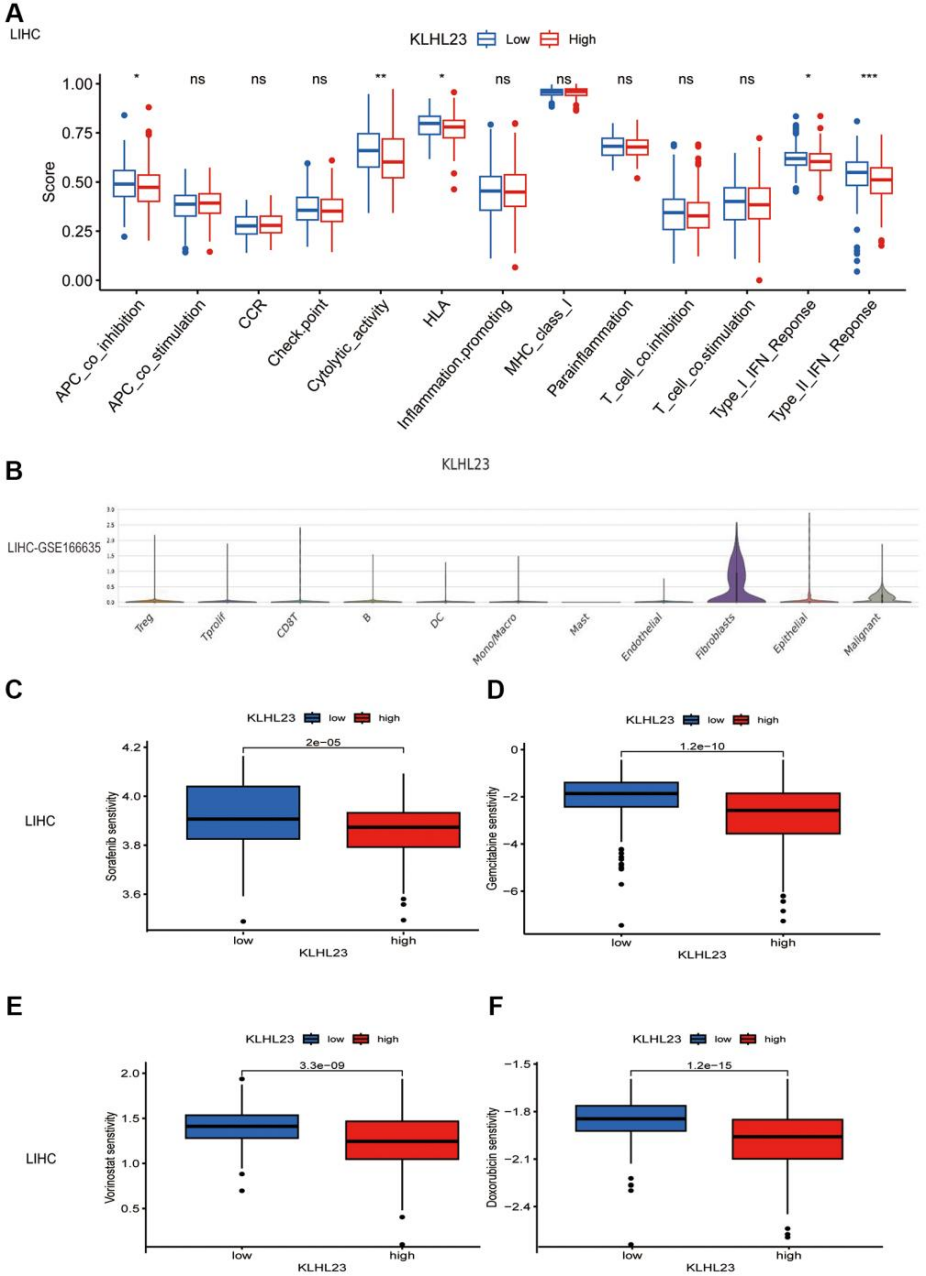
**The functional role of KLHL23 in LIHC.** (**A**) Differences in the immune cell signaling pathway in different proportions of immune cells between groups with high and low KLHL23 expression in LIHC. (Abbreviation: ns: no significant difference. ^*^P < 0.05; ^**^P < 0.01; ^***^P < 0.001; ^****^P < 0.0001). (**B**) Single cell analysis of KLHL23 expression in different cell types. (**C**–**F**) Analysis of drug sensitivity based on KLHL23 expression in LIHC. (**C**) Sorafenib, (**D**) Gemcitabine, (**E**) Vorinostat, and (**F**) Doxorubicin. ^*^P < 0.05; ^**^P < 0.01; ^***^P < 0.001; ^****^P < 0.0001).

Comparing the effects of KLHL23 expression on immune pathways, we observed that both type II IFN response and cytolytic activity were decreased in the high KLHL23 expression group, indicating that KLHL23 affects the anti-tumor activity of the tumor immune microenvironment. (Figure 7A and Supplementary Figure 2A–2P). Utilizing the single-cell database (GSE166635), we observed prominent KLHL23 expression in fibroblasts and malignant cells (Figure 7B). To further investigate the biological consequences of KLHL23 expression in malignant cells, we conducted a gene set variation analysis (GSVA) using multiomic data sourced from TCGA. Our analysis revealed significant enrichment in pathways related to immune responses and the EMT (Supplementary Figure 3).

In specific cancer contexts, such as LIHC, KLHL23 expression exhibited a negative association with the IL6-JAK-STAT3 signaling pathway, but a positive association with the G2M checkpoint and MYC gene targeting signaling pathway. These observations shed light on the regulatory mechanisms and the tumor microenvironment’s influence within the context of LIHC (Supplementary Figure 3). GO enrichment pathways in the KLHL23 high expression group across cancers were similar and could be classified into three categories: immune response and T cell activation pathways, extracellular matrix adhesion pathways, and RNA splicing and ion transport pathways (Supplementary Figure 3).

Furthermore, we evaluated the sensitivity of potential drugs in relation to KLHL23 expression levels in LIHC using the pRRophetic package (Figure 7C–7F). Our study demonstrated that reduced KLHL23 expression was associated with increased responsiveness to chemotherapy drugs such as sorafenib, gemcitabine, vorinostat, and doxorubicin, which are commonly used in LIHC treatment. This suggests a potential role for KLHL23 in determining drug sensitivity in LIHC therapy. However, these findings are based on *in silico* data analysis and require further validation through experimental studies and clinical trials.

## DISCUSSION

Previous studies have suggested the potential involvement of KLHL23 in cancer development, with some focusing on specific aspects such as genetic mutations or expression levels [2, 5, 6, 19]. Our research expands upon this foundation by examining KLHL23 across multiple dimensions, including methylation status, mutations, clinical outcomes, and immune cell infiltration in a wide range of cancers.

This research provides deep insight into the role of KLHL23 in the progression of various cancers. KLHL23 expression levels in various cancers are influenced by a variety of factors, including genetic alterations such as copy number variations and mutations. Regulatory mechanisms, including the activity of transcription factors and enhancers, also contribute to these variations. Additionally, epigenetic modifications are known to affect KLHL23 expression in different cancer types. Furthermore, signaling pathways and the characteristics of the tumor microenvironment are also critical in modulating KLHL23 expression. In particular, the association between KLHL23 alterations and clinical prognosis has been of interest in previous studies, but our research took this a step further by linking these alterations to the extent of immune cell infiltration, suggesting a possible immunomodulatory role of KLHL23 in the tumor microenvironment. The identification of KLHL23 as a critical biomarker and potential therapeutic target, particularly in HCC, is a significant step forward. Previous studies have suggested the relevance of KLHL23 in HCC [2], but our findings solidified its role and suggest new avenues for targeted therapy. In particular, our investigations have identified KLHL23 as a critical biomarker and potential therapeutic target, especially in the context of HCC. Overall, the findings of our study shed new light on the diverse functions of KLHL23 across a spectrum of cancers, paving the way for future research and potential clinical applications. In discussing our findings, while we have uncovered crucial aspects of KLHL23’s involvement in cancer, many questions remain unanswered. The exact mechanisms by which KLHL23 influences tumor progression require further investigation, particularly in the context of different cancer types. Additionally, the potential of KLHL23 as a therapeutic target warrants in-depth exploration, including the development of targeted therapies and understanding potential resistance mechanisms.

In conclusion, our research not only confirms previous studies, but also provides new insights into the diverse functions of KLHL23 in cancer. This provides a solid foundation for future research that may lead to significant advances in cancer diagnosis, prognosis, and therapy, especially in the field of personalized medicine.

## Supplementary Materials

Supplementary Figures
