# Supplementary material for: Unveiling KLHL23 as a key immune regulator in hepatocellular carcinoma through integrated analysis
Source: Aging (Albany NY). 2024 Dec 4;16(22):13608–26. doi: 10.18632/aging.206167 (PMC11723656; doi:10.18632/aging.206167)
Supplement: Supplementary Figures [file aging-16-206167-s001.pdf]

SUPPLEMENTARY FIGURES

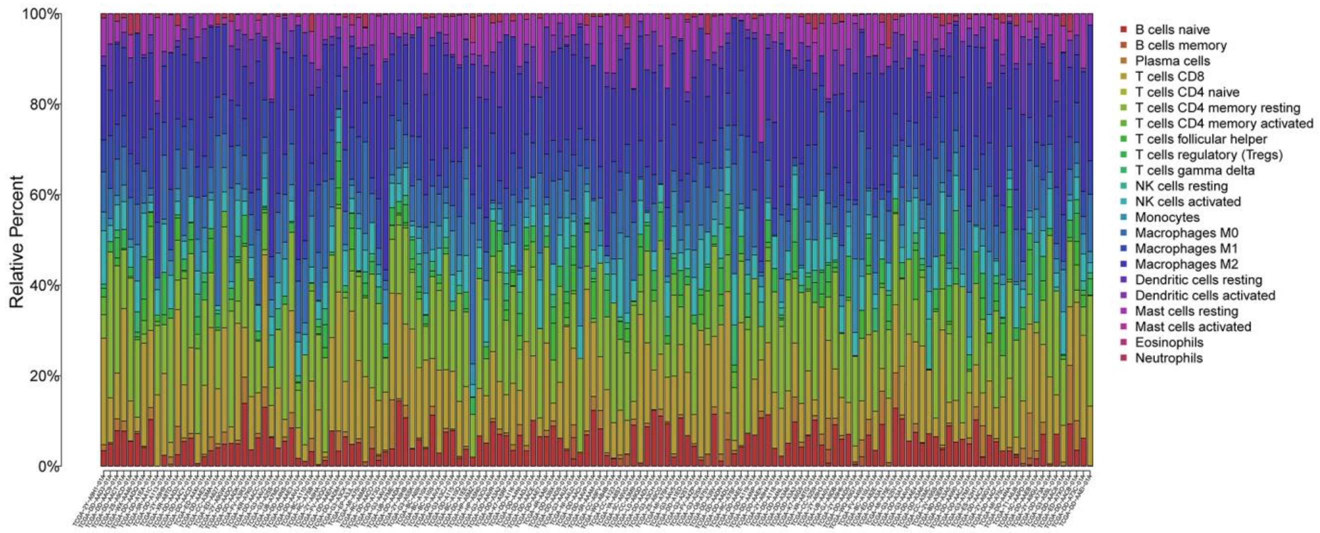

Supplementary Figure 1. The role of KLHL23 in the tumor microenvironment of LIHC. Immune cell distribution in KLHL23 lower expression LIHC patients.

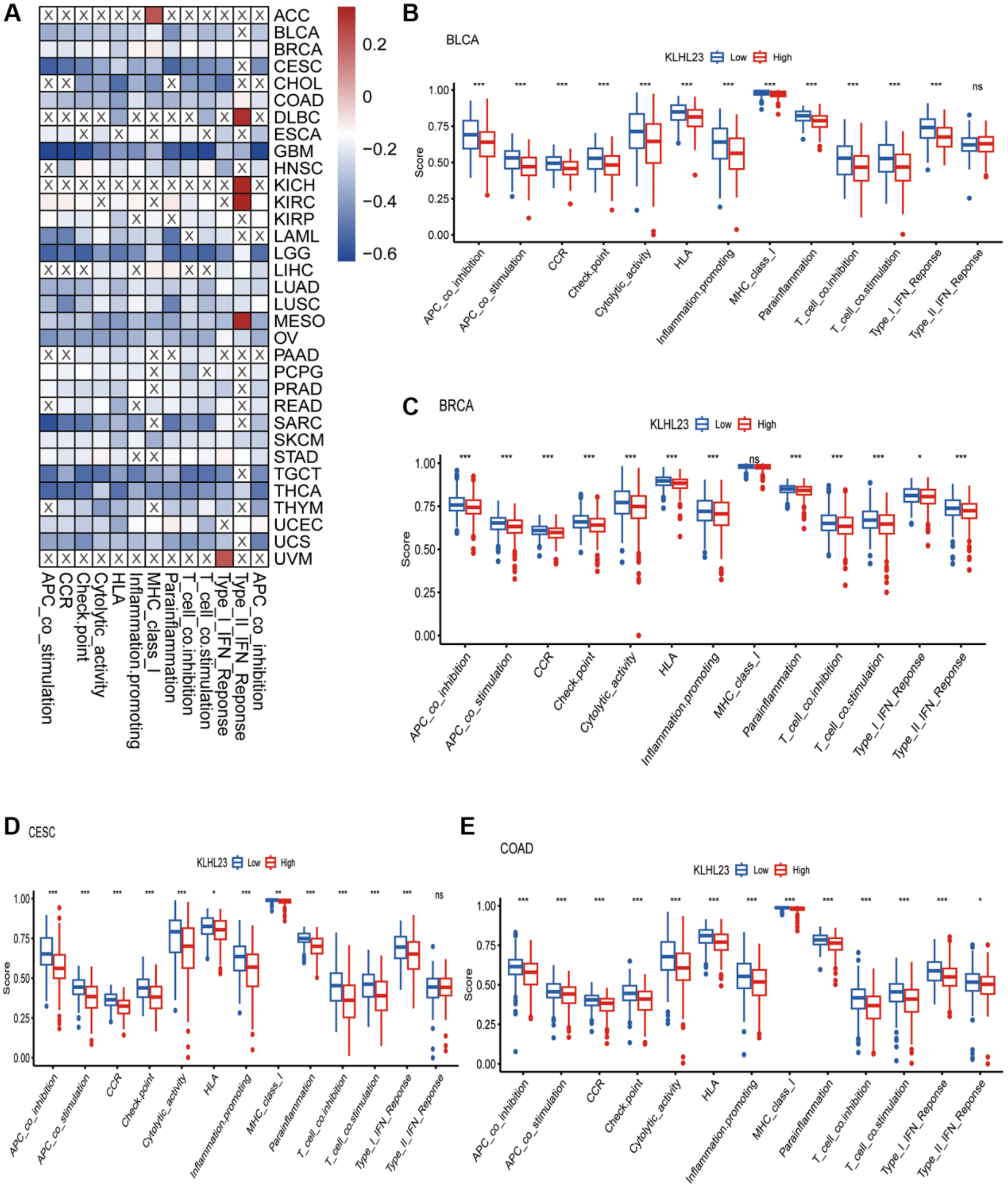
A
B
BLCA
C
BRCA
D
CESC
E
COAD
KLHL23 Low High
Score
0.2
0
−0.2
−0.4
−0.6
ACC
BLCA
BRCA
CESC
CHOL
COAD
DLBC
ESCA
GBM
HNSC
KICH
KIRC
KIRP
LAML
LGG
LIHC
LUAD
LUSC
MESO
OV
PAAD
PCPG
PRAD
READ
SARC
SKCM
STAD
TGCT
THCA
THYM
UCEC
UCS
UVM
APC_co_inhibition
APC_co_stimulation
CCR
Check.point
Cytolytic_activity
HLA
Inflammation.promoting
MHC_class_I
Parainflammation
T_cell_co.inhibition
T_cell_co.stimulation
Type_I_IFN_Reponse
Type_II_IFN_Reponse

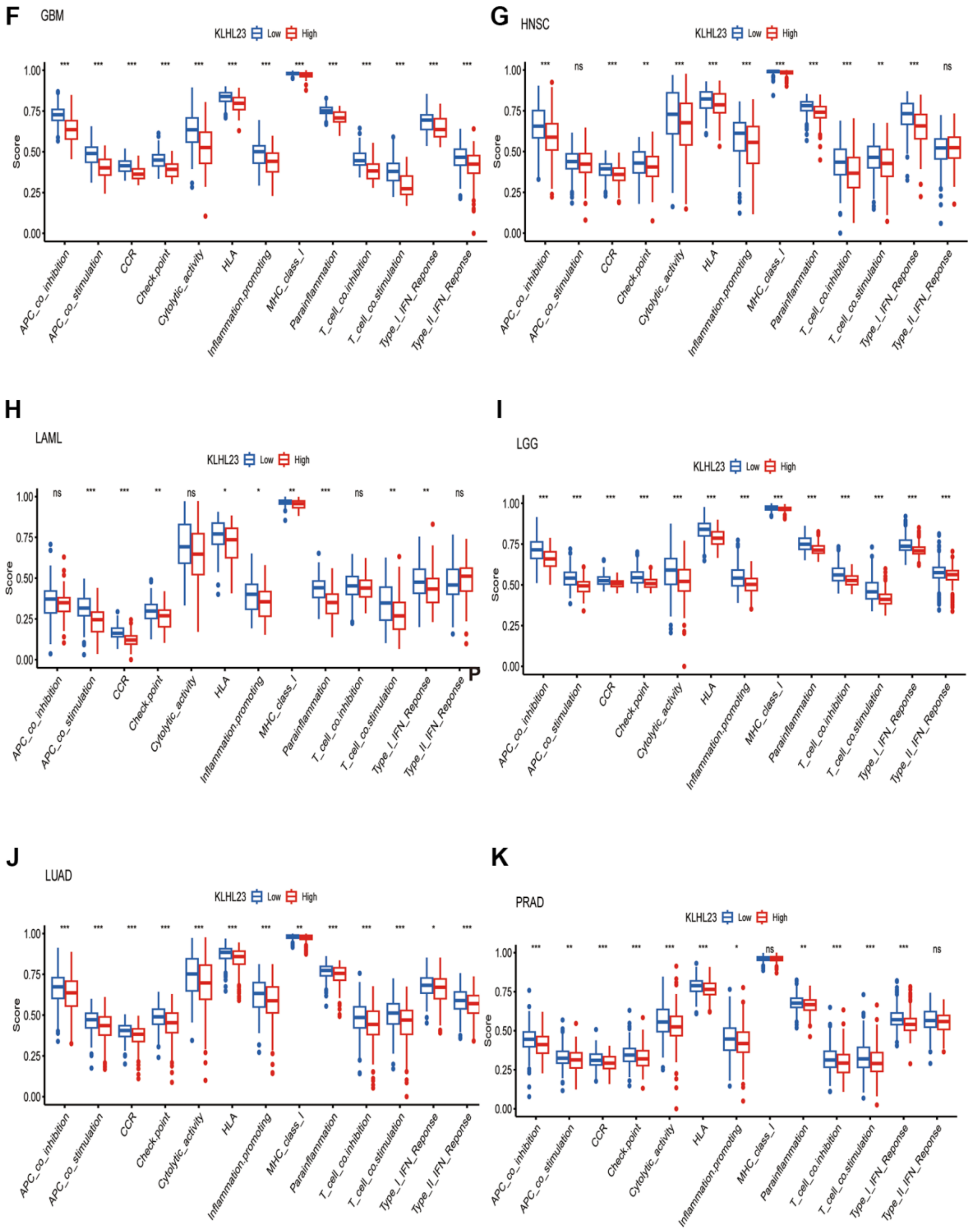
F
GBM
G
HNSC
H
LAML
I
LGG
J
LUAD
K
PRAD
KLHL23 Low High
Score
1.00
0.75
0.50
0.25
0.00
APC_co_inhibition
APC_co_stimulation
CCR
Check.point
Cytolytic_activity
HLA
Inflammation.promoting
MHC_class_I
Parainflammation
T_cell_co.inhibition
T_cell_co.stimulation
Type_I_IFN_Reponse
Type_II_IFN_Reponse

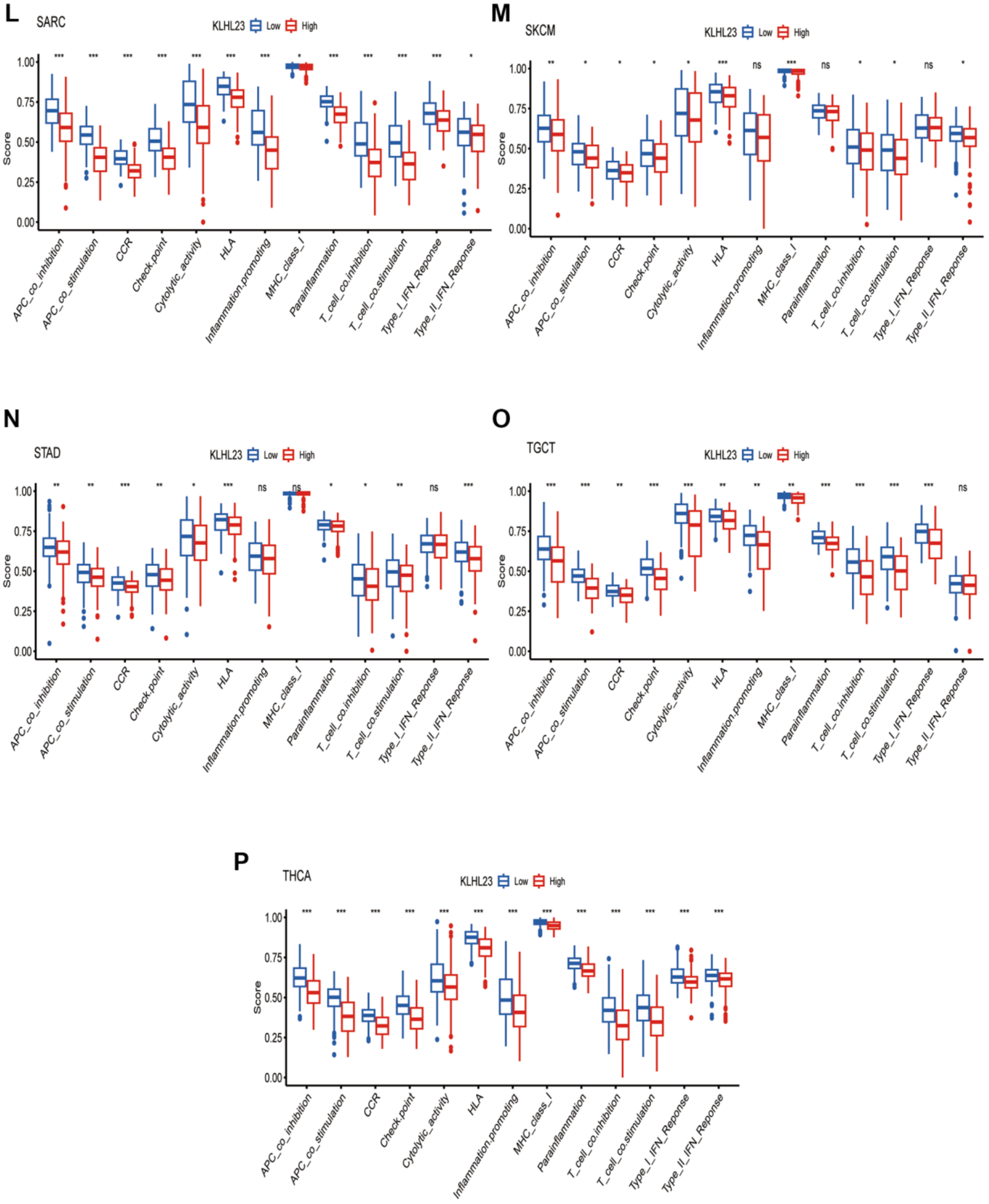

Supplementary Figure 2. The effect of KLHL23 on immunological status across cancers. (**A**) Association between KLHL23 and 13 tumor-associated immune cells, as calculated using the ssGSEA algorithm. (**B**–**P**) Differences in the various immune cell proportions between groups with high and low KLHL23 expression in representative cancers. (Abbreviation: ns: no significant difference. $^{*}P < 0.05$; $^{**}P < 0.01$; $^{***}P < 0.001$; $^{****}P < 0.0001$).

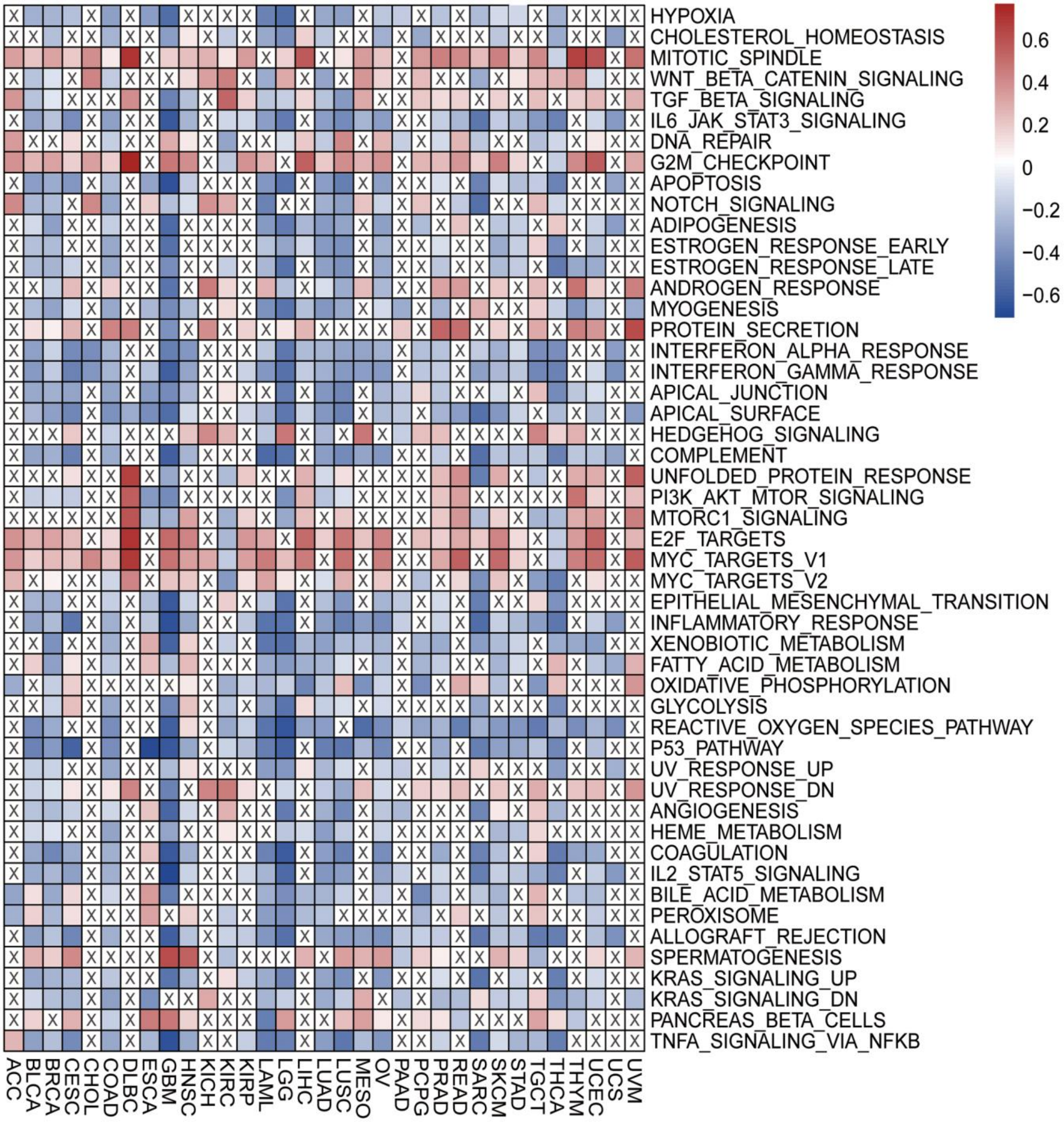

Supplementary Figure 3. KLHL23 signaling pathway analysis. Single cell analysis of KLHL23 expression in different cell types.
